# Rapid determination of hippuric acid as an exposure biomarker of toluene using a colorimetric assay and comparison with high-performance liquid chromatography

**DOI:** 10.1038/s41598-024-59641-z

**Published:** 2024-04-30

**Authors:** Fatemeh Dehghani, Saeed Yousefinejad, Nabiollah Mobaraki, Mohsen Nekoeinia, Bahram Hemmateenejad

**Affiliations:** 1 Student Research Committee, Shiraz University of Medical Sciences, Shiraz, Iran; 2 Department of Occupational Health Engineering, School of Health, Shiraz University of Medical Sciences, Shiraz, Iran; 3 Institute of Medicinal and Pharmaceutical Chemistry, University of Technology Braunschweig, Braunschweig, Germany; 4 Soil and Water Research Department, Isfahan Agricultural and Natural Resources Research and Education Center, AREEO, Isfahan, Iran; 5 Chemistry Department, Shiraz University, Shiraz, Iran

**Keywords:** Colorimetric, Occupational assessment, Central composite design, Exposure, Biomonitoring, Aromatic compounds, Environmental sciences, Health occupations, Chemistry

## Abstract

Occupational exposure to toluene is associated with health risks that require reliable monitoring methods. Hippuric acid (HA), a urinary metabolite of toluene, serves as a valuable biomarker for such exposure. Colorimetric methods for the quantitative determination of HA have gained prominence due to their simplicity, cost-effectiveness, and suitability for field application. In the present study, a simple colorimetric technique was optimized for the determination of HA in the urine sample, and compared with a usual HPLC technique. The central composite design (CCD) was applied to examine the effective parameters on the colorimetric determination of HA. The calibration curve for HA was established within the concentration range of 6 to 100 mg L^−1^ with R^2^ = 0.97. The detection limit (LOD) and quantification limit (LOQ) were determined to be 1.8 mg L^−1^ and 6 mg L^−1^ respectively. The relative standard deviation (RSD%) was less than 5%, and the recovery% (R%) was 90.5–100.1. The overall results showed good agreement between the colorimetric and HPLC results. There was a significant relationship between the results obtained from HPLC and colorimetric methods especially for higher concentration levels of HA (≥ 500 mg/g creatinine). In conclusion, our optimized colorimetric method is a simple, cost-effective, and rapid method for determination of HA in occupational exposure, which is comparable with the HPLC technique.

## Introduction

Despite implementing different administrative and engineering control measures to mitigate exposure to chemical agents, a significant portion of the human population continues to be in contact with volatile organic compounds (VOCs) in living and occupational environments^1^. Toluene is one of the most VOCs widely consumed products in various industrial processes and environmental activities. Workers in industries such as painting, rubber manufacturing, chemical production, and petroleum refining may be exposed to toluene^2,3^. Environmental exposure to toluene can also occur through vehicle emissions, industrial emissions, and the evaporation of solvents. Following exposure to toluene a wide range of adverse health effects can occur depending on factors such as concentration, duration, and route of exposure^4–9^. Therefore, it is important to evaluate exposure for conducting an accurate health risk assessment. Biological monitoring is one of the critical components of any exposure assessment program because it provides a direct assessment of the internal dose of exposed populations^10^. It measures the actual amount of a substance that has been absorbed, metabolized, and accumulated in the body, and it is performed by measuring specific biomarkers or metabolites of the target compound. Inhalation exposure to toluene triggers a series of metabolic reactions in the human body, leading to the formation of various metabolites^11,12^. One of the major metabolic pathways involves the conversion of toluene to hippuric acid (HA) with the maximum allowable level of 1.6 g/g creatinine^13^. Several analytical methods have been developed to quantify HA in biological specimens, each with its own set of advantages and limitations^14–17^. These methods were mostly developed based on solid-phase and liquid-phase microextraction. The solid phase-based techniques include dispersed magnetic layered double hydroxides, microextraction by packed sorbent (MEPS) using Fe_3_O_4_@TbBd nanobeads, and the use of different molecularly imprinted nanoparticles^18–20^. Despite many advantages of the proposed methods such as selectivity and specificity, they suffer from limitations. These involve multiple steps (conditioning, loading, washing, elution), making them more intricate than some other methods. The solid cartridges and sorbents can be expensive. They also use organic solvents, which can be environmentally concerning. Various liquid phase microextraction (LPME) techniques such as, dispersive liquid–liquid microextraction by novel materials such deep eutectic solvents, hollow fiber supported liquid membrane, ion-pair-based hollow-fiber liquid phase micro extraction have also been developed for quantification of HA in urine sample^21–23^. Despite all advantages, these methods have inherent limitation, such as low extraction efficiency, sensitivity to matrix effects, and use of toxic extraction solvents.

All the above-mentioned techniques were coupled with high-performance liquid chromatography (HPLC) and gas chromatography (GC). These methods necessitate advanced instrumentation, intricate operational procedures, and extended analysis durations. However, it is crucial to employ simple and rapid methods for assessing occupational and environmental exposure. The colorimetric techniques based on spectroscopic analysis offer a straightforward and cost-effective alternative to chromatographic methods. A total of five methods were developed for colorimetric analysis of HA in urine samples. The first colorimetric technique for HA determination was proposed in 1908, and several colorimetric reagents have been proposed over time^24–27^. Despite the simplicity of the proposed methods, a notable limitation arises from their time-consuming nature, extending the analysis duration up to several hours. A slightly faster method has been developed in previous reports, in which benzene sulfonyl chloride acted as a reagent for the detection of HA with an analysis time of 1.5–2 h^28^. A recently published study to quantify HA in human urine replaced benzene sulfonyl chloride with solid *p*-toluenesulfonyl chloride (p-Tscl) to decrease the time of the reaction. This assay could detect HA in urine in 2min. However, this assay used NMR spectroscopy for quantification of HA. The limit of detection was also reported as 10 mg L^−1^, which may be insufficient for measuring HA in low occupational exposure levels^29^. The present study aimed to develop and optimize a simple method for rapid determination of HA as an exposure biomarker of toluene and compare it with a conventional HPLC method. In the present study, we tried to overcome the limitations of the previous methods to obtain an optimum analysis time with an acceptable LOD for occupational exposure monitoring of HA. The central composite design was applied to obtain experimental conditions for a simple, fast, and sensitive analysis of HA determination by a less sophisticated instrument like spectrophotometry. By optimizing the appropriate amount of solvents and reagents, the colorimetric reaction can occur so fast in an acidic environment. In such conditions, the reaction kinetic is favorable, and the rate of product formation is maximum. To ensure accuracy and confidence in the optimized method for toluene exposure assessment, we conducted a comparison with a routine HPLC technique.

## Materials and methods

### Reagents and solutions

Hippuric acid (Analytical grade), benzene sulfonyl chloride (BSC), and pyridine were obtained from Sigma-Aldrich. Methanol (HPLC grade), hydrochloric acid (HCl), and sodium hydroxide were supplied from Merck Company. Doubled distilled water was used from a milli-Q system (Bedford, USA). Standard solutions of HA (1000 mg L^−1^) were obtained using double-distilled water. The daily standards were prepared from the stock solution. The prepared stock solution was stored in the refrigerator for 10 days. The stability of the stock solution was checked by preparing a solution with a specific concentration of the analyte and comparing the results of absorbance during the time.

### Colorimetric method

**Figure 1:**
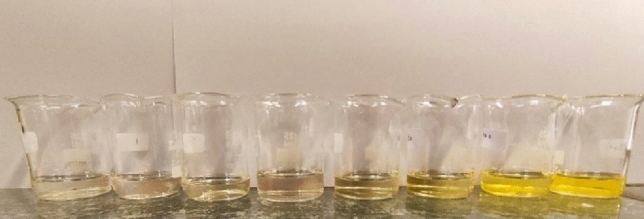
The urine samples containing different concentrations of HA (6.0–100 mg L^−1^).

For colorimetric determination of HA, 5 mL of urine sample was placed into a glass tube and then centrifuged for 5 min at 4000 rpm. Then, the supernatant part was filtered by a 0.45 μm filter, and 2.5 mL of the solution was diluted in a 1:5 ratio with double distilled water. For the determination of analytical conditions, an appropriate amount of BSC (A) was added to the urine sample (0.2 ml), and subsequently mixed with a specific amount of pyridine (B). Then, the pH of the solution was adjusted in the defined value (E). After the incubation period (D) and the formation of a yellow-colored complex, an appropriate amount of methanol (C) was added to stop the reaction. The absorbance of the colored solution was recorded at the wavelength of maximum absorption (λ_max_) at 410 nm using a spectrophotometer (Carry 60 UV–vis spectrophotometer- Agilent, USA). A color chart for the quantification of HA acid was plotted using standard samples containing 6.0–100 mg L^−1^ HA. Figure 1 shows the urine samples containing different concentrations of HA.

### HPLC method

For analyzing the sample with the HPLC technique, the typical liquid liquid microextraction method was applied^30^. In summary, 1.0 mL of a diluted urine sample (1:5 ratio) was placed into a 15-mL glass tube and subsequently mixed with 80 µL of HCl (6 N) and 0.3 g of sodium chloride. Then 4 mL ethyl acetate was added and mixed for 2 min by rotation. After centrifugation at 4000 rpm for 5 min, 200 µL of the supernatant part of the solutions was collected into an HPLC vial and evaporated using a heated water bath (40 ℃) and a stream of nitrogen. Then, the residue in re-dissolved in 200 µL of distilled water, and 20 µL was injected into HPLC. The HPLC instrument (Knauer, Germany), consisted of a high-pressure pump (K-1001 series), and a UV detector was used. The chromatographic analysis was done on a C18 column (250 mm × 4.6 mm × 5 μm). The mobile phase was a mixture of methanol containing 0.1% acetic acid with a flow rate of 1.0 mL min^−1^. The detector’s wavelength was set at 225 nm. To create the calibration curve, solutions of HA range from 6.0 to 100 mg L^−1^ was prepared by spiking a suitable amount of HA in urine.

### Real sample collection

Metabolite-free urine samples were collected from volunteers. Furthermore, to evaluate the correlation of two colorimetric and HPLC methods. 10 urine samples were taken from workers in a petrochemical industry at the end of the shift. All urine samples were kept at – 20 °C before analysis. The procedures of urine sampling and human participation were performed in accordance with the ethical principles in the Helsinki declaration. The study was also approved by the Ethics Committee of the Shiraz University of Medical Sciences.

### Experimental design

Several parameters were investigated to optimize the efficiency of the colorimetric reaction. The key parameters included the amount of BSC, the volume of pyridine, the solution pH, the volume of methanol, and incubation time. To achieve this optimization, we employed an experimental design methodology^31,32^. Response Surface Methodology (RSM) was utilized in this study, employing a Central Composite Design (CCD) with five variables: volume of BSC (A, mL), volume of pyridine (B, mL), volume of methanol (C, mL), incubation time (E, min), and pH (E). The experimental data obtained from the CCD were then fitted to a polynomial equation to predict the colorimetric response.

The polynomial equation used for prediction was as follows (Eq. 1):

1 $$Y={b}_{0}+\sum \limits_{i=1}^{n}{b}_{i}{x}_{i}+ \sum \limits_{i=1}^{n}{b}_{ii}{x}_{i}^{2}+ \sum \limits_{i<j}^{n}{b}_{ij}{x}_{i}{x}_{j}+\varepsilon$$

where Y represents the predicted colorimetric response, n is the number of parameters (in this case, five), x_i_ and x_j_ are the independent factors, b_0_, b_i_, b_ii_, and b_ij_ are described as the regression coefficients, and ε represents the residual error.

To assess the reliability of the polynomial equation and its fit to the response, we conducted an Analysis of Variance (ANOVA) by using Design Expert software (11.0.8). The determination coefficient (R^2^) was applied to evaluate the fitting between the polynomial equation and the experimental data. Additionally, the significance of the polynomial equation terms was evaluated by calculating the F value at P < 0.05.

### Ethics declarations

The manuscript has been approved by the ethics committee of Shiraz University of Medical Sciences (Ethical ID: IR.SUMS.REC.1400.038). Informed consents were obtained from all subjects for urine collection and analysis.

## Results and discussion

In the present study, a colorimetric technique was optimized for the determination of HA from urine samples, and compared with the HPLC technique. The yellow color formed in the reaction of BSC and pyridine with HA is likely due to the formation of a colored compound of benzoyl HA.

The color of urine samples from unexposed persons was colorless and transparent. However, to prevent potential color interference between the natural yellow of the urine sample and the color change induced by the analytical reagents, a dilution factor of fivefold (1:5 dilution) was employed. This resulting dilution allowed for the accurate quantification of the analyte of interest without compromising the integrity of the analytical method. It is important to note that all subsequent calculations and results were adjusted to account for this dilution factor, ensuring the accuracy and reliability of the analytical measurements.

### Mechanism of the colorimetric reaction

**Figure 2:**
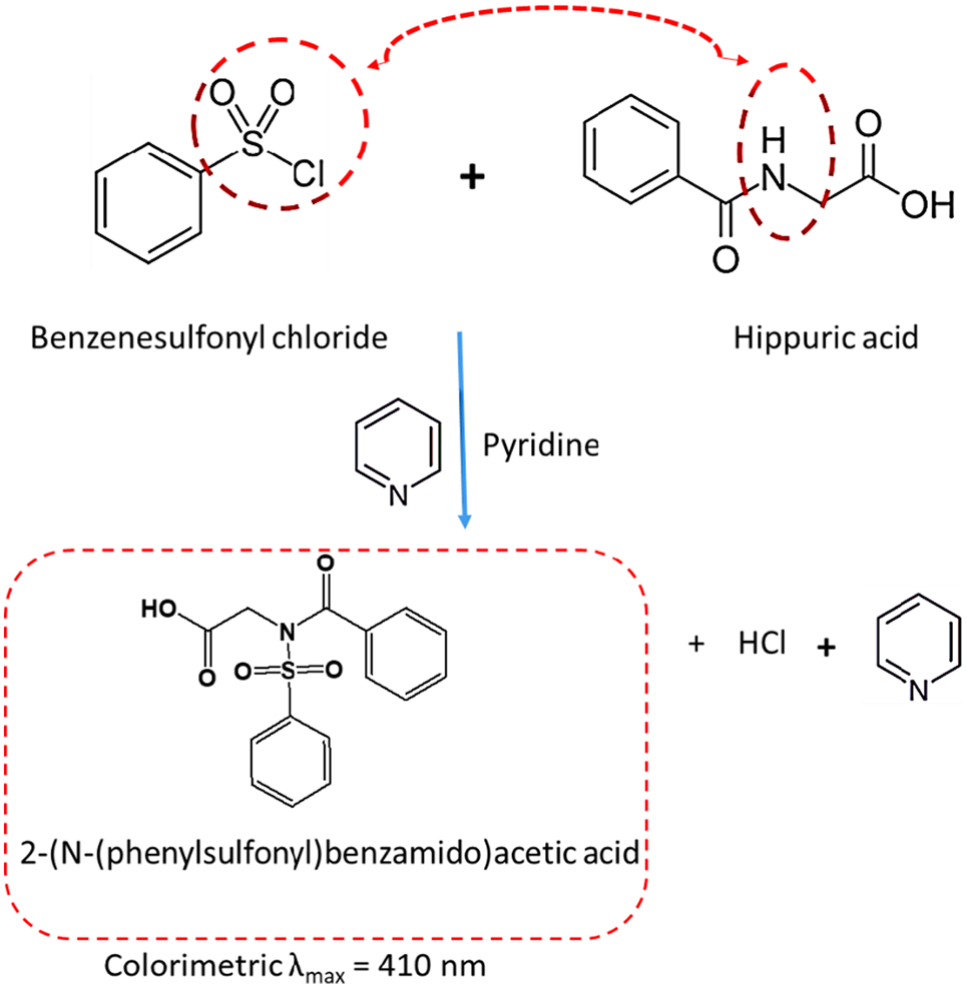
Reaction scheme for the color reaction of HA.

The chemistry of the HA colorimetric assay involves the reaction between benzenesulfonyl chloride (C_6_H_5_SO_2_Cl) with hippuric acid (C_9_H_9_NO_3_) in the presence of a base (typically pyridine). The amino group (–NH_2_) of HA attacks the sulfonyl chloride group, leading to the formation of 2-N-phenylsulfonyl benzamide acetic acid. This product creates a chromophore, leading to the absorption of visible light and the observed color change. The colorimetric reaction is shown in Fig. 2.

### Central composite design for optimization

The results of the experimental design are shown in Table 1. As shown, a total of 30 runs was designed to achieve the optimized values of parameters. The results of Central Composite Design (CCD) with five variables and their levels are shown in Table 2.

**Table 1:** The matrix of central composite design and responses.

Run	A	B	C	D	E	Absorbance
1	0.6	0.1	2	14	5.5	0.803
2	0.6	0.3	2	1.3	5.5	0.593
3	0.8	0.4	3	21	8	0.463
4	0.4	0.2	1	7	8	0.369
5	0.6	0.3	2	14	5.5	0.599
6	0.4	0.4	1	7	3	1.165
7	0.6	0.3	2	14	5.5	0.932
8	0.6	0.3	0.2	14	5.5	0.671
9	0.8	0.2	3	7	8	0.397
10	0.25	0.3	2	14	5.5	0.588
11	0.4	0.2	3	7	3	0.952
12	0.4	0.2	1	21	3	1.020
13	1	0.3	2	14	5.5	0.739
14	0.6	0.3	3.8	14	5.5	0.949
15	0.8	0.4	1	21	3	1.260
16	0.4	0.4	3	7	8	0.566
17	0.6	0.3	2	14	1	1.249
18	0.6	0.3	2	14	5.5	0.930
19	0.6	0.3	2	14	5.5	0.644
20	0.8	0.4	3	7	3	1.168
21	0.8	0.4	1	7	8	0.242
22	0.4	0.4	1	21	8	0.599
23	0.6	0.3	2	14	5.5	0.966
24	0.6	0.3	2	26.7	5.5	0.800
25	0.6	0.5	2	14	5.5	0.814
26	0.6	0.3	2	14	10	0.329
27	0.4	0.2	3	21	8	0.645
28	0.8	0.2	1	21	8	0.550
29	0.4	0.4	3	21	3	0.988
30	0.6	0.3	2	14	5.5	0.843

**Table 2:** The matrix of central composite design and responses.

Variables	Description	Levels of variables				
		−α	−1	0	1	+ α
A	BSC volume (mL)	0.25	0.4	0.6	0.8	1
B	Pyridine volume (mL)	0.1	0.2	0.3	0.4	0.5
C	Methanol volume (mL)	0.2	1	2	3	3.8
D	Incubation time (min)	1.3	7	14	21	26.7
E	pH	1^a^	3	5.5	8	10

^a^pH was adjusted as low as possible.

In the experiment, we took measures to mitigate the impact of uncontrolled variables by randomly arranging the experimental runs within the CCD matrix, as detailed in Table 3. After conducting an ANOVA analysis of the data collected, we found that a quadratic polynomial model was a good fit for the data. To refine the model and eliminate parameters or interaction terms with non-significant P-values (> 0.1), we performed a backward elimination factor selection. The resulting equation from this process was as follows (Eq. 2):

2 $$\begin{aligned} {\text{Absorbance}} & = {361328}.{7} - {1}0{2744 } \times {\text{ A}} + {76144}.{58 } \hfill \\ & \quad \times {\text{ B}} + {55792 } \times {\text{ C}} + {19513}.{25 } \times {\text{ D }} + { 74675}.{67 } \times {\text{ E}} \hfill \\ & \quad + {151962}.{3 } \times {\text{ AC}} + {298752}.{8 } \times {\text{ AE }} + { 77272}.{88 } \times {\text{ CD }} \hfill \\ & \quad + {198722}.{8 } \times {\text{ CE}} + {324531}.{8 } \times {\text{ E}}^{{2}} . \hfill \\ \end{aligned}$$

**Table 3:** Analysis of variance (ANOVA), model statistics summary, and quality of the quadratic model.

Source of variation	Sum of squares	DF	Mean square	F-value	P-value	Importance
Model	2.203	7	0.315	27.013	< 0.0001	Significant
A-BSC volume	0.006	1	0.006	0.514	0.4804	
C-Methanol Volume	0.006	1	0.006	0.502	0.4855	
D-Time	0.038	1	0.038	3.275	0.0829	
E-pH	1.993	1	1.993	171.029	< 0.0001	
AE	0.083	1	0.083	7.085	0.0137	
CE	0.037	1	0.037	3.137	0.0893	
DE	0.041	1	0.041	3.551	0.0717	
Residual	0.280	24	0.012			
Lack of fit	0.153	19	0.008	0.319	0.9685	Not significant
Pure error	0.126	5	0.025			
Cor total	2.483	31				
Model statistics						
R^2^ (regression coefficient)		R^2^ (adjusted)		R^2^ (predicted)		Adequate precision
0.887		0.855		0.828		20.019

**Figure 3:**
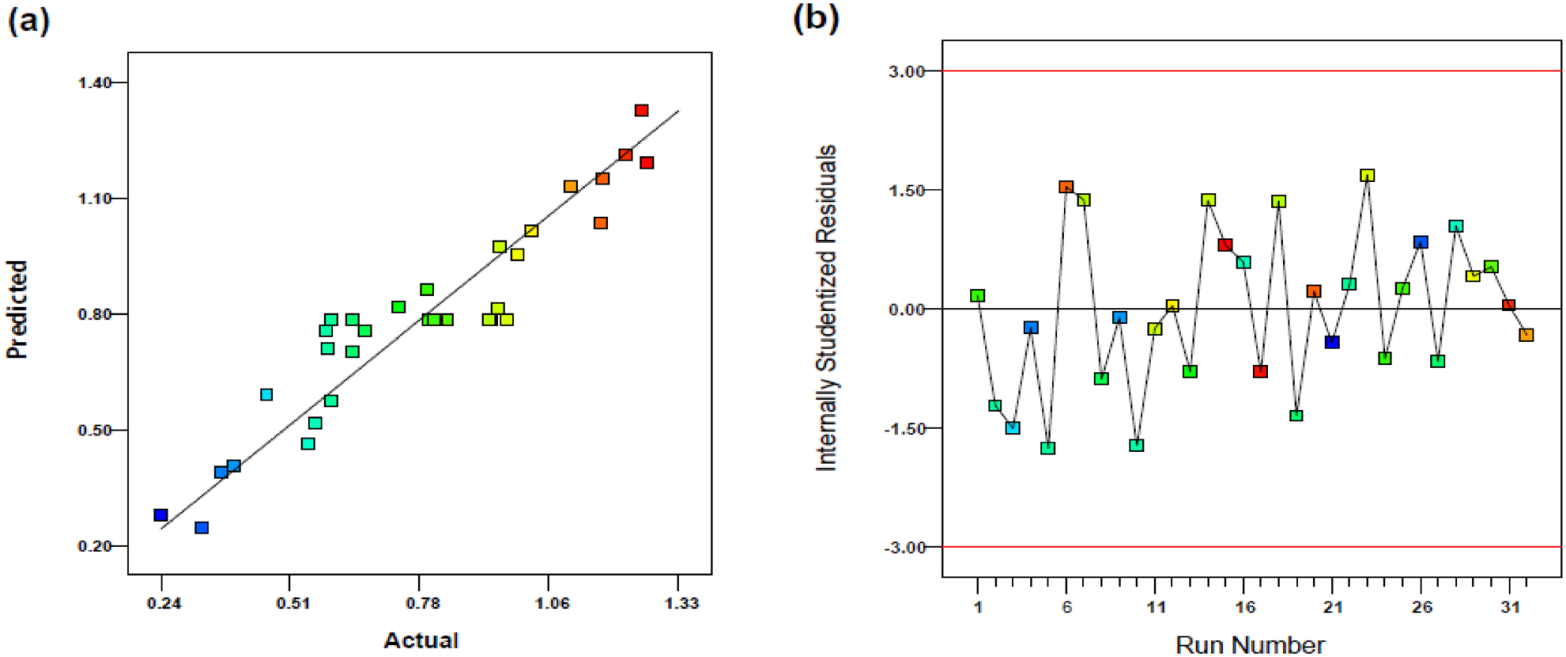
The predicted value vs. actual response (**a**); and the internally studentized residuals vs. performed runs (**b**).

To assess the significance of the model, we employed Fisher's statistical test (F-test). The calculated F-value for our model was 27.013, surpassing the critical F-value for the required degree of freedom, demonstrating the model's significance as shown in Equation. Another important criterion for validating multiple linear regression (MLR) models from the experimental design table is the Lack of Fit (LOF) test. In our case, the LOF F-value was 0.319, indicating that LOF was not significant, and no pure error was observed in the suggested model. Furthermore, to examine the fitness and predictive capability of our model, we computed calibration R^2^ (R^2^_cal_), prediction R^2^ (R^2^_pred_), and adjusted R^2^ (R^2^_adj_). As displayed in Table 2, R^2^_cal_ indicated that our model accounted for 88.7% of the data. Additionally, R^2^_adj_ exceeded 0.8, confirming its goodness of fit based on the literature. Moreover, R^2^_pred_ (= 0.828) closely matched R^2^_adj_ (= 0.855), with a difference of less than 0.03, signifying excellent prediction ability (Table 3). All of these metrics indicate a strong correlation among the variables and interaction terms in Eq. (2) with the absorbance as the response for the determination of HA by colorimetric method. To demonstrate the model's goodness of fit, we provided a plot of predicted absorbance as the response versus the experimental values in Fig. 3a. Furthermore, to verify the applicability of our model, we examined the residual values (the difference between actual and predicted responses) within ± 3σ, as indicated in Fig. 3b. The studentized residual of our model fell within ± 2σ, indicating model reliability. Additionally, the residual values for all runs were scattered randomly on two sides of the zero, confirming the lack of systematic errors.

### Optimization of effective variables using CCD

Based on the findings presented in Eq. (2), the multiparameter model introduced various interaction terms, encompassing interactions among factors (AE, CE, and DE). To illustrate the combined effects of these factors, we employed three-dimensional (3D) response surface curves. These curves offer valuable insights into the interplay of factors within the interaction terms, aiding in the discovery of optimal values for independent parameters. Furthermore, these curves reveal critical details about the highest achievable response and potential interactions between two independent factors.

**Figure 4:**
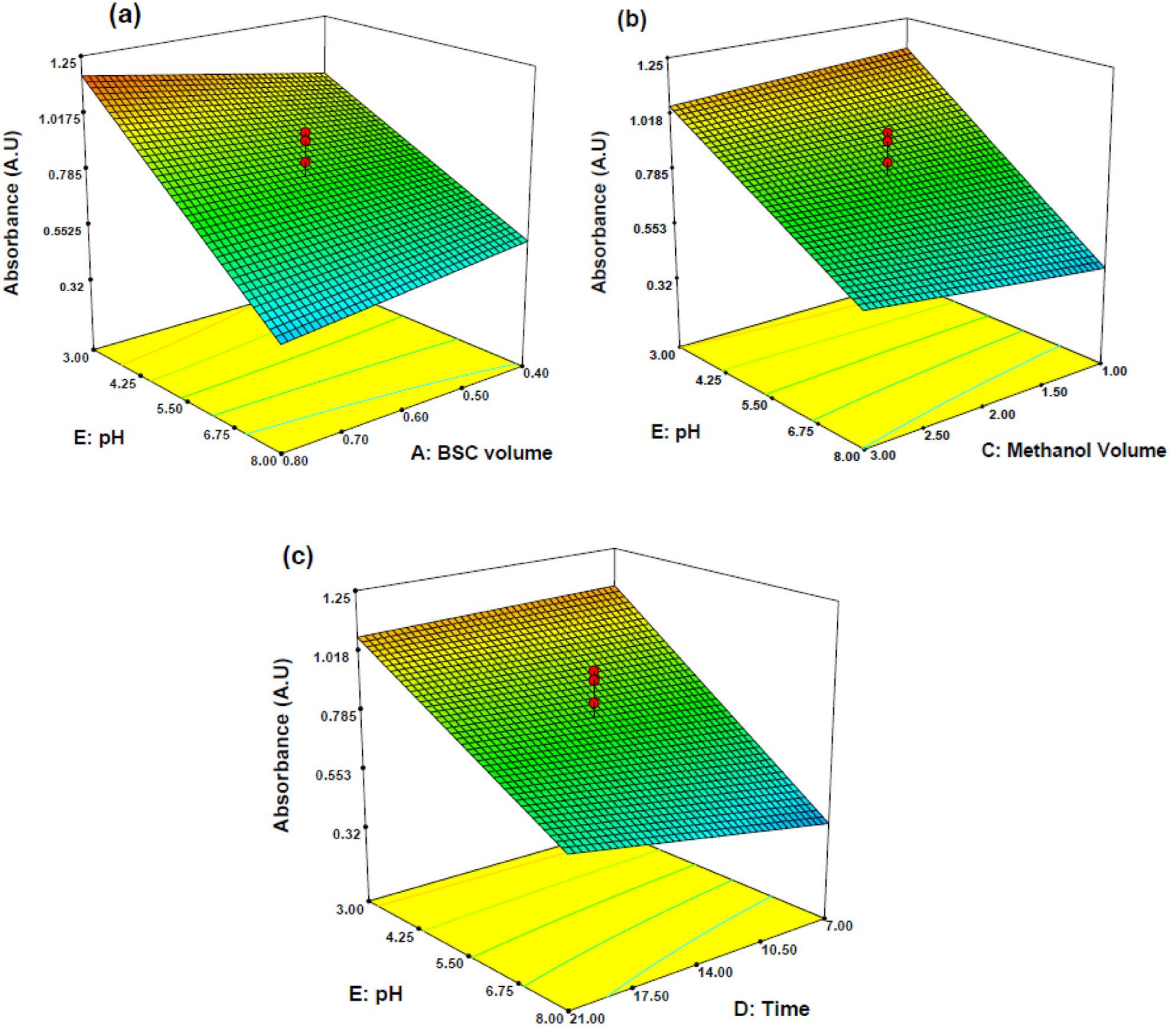
Three-dimensional plots of interaction effects of independent variables: pH-BSC volume (**a**), pH-methanol volume (**b**), pH-time (**c**) for determination of HA in the urine sample.

Figure 4a shows the effect of pH and BSC volume on the response (absorbance). As illustrated, the maximal absorbance was achieved at the pH of 4.25, and absorbance decreased by increasing the pH and decreasing the BSC volume. BSC is a reagent and reacts with HA to form a compound known as benzoyl hippuric acid. The reaction involves the substitution of the chlorine atom in BSC with the amino group in hippuric acid. The benzoyl hippuric acid formed in the reaction is often colored or can be converted into a colored derivative through subsequent reactions. The specific color and intensity depend on the conditions and reagents used in the colorimetric assay. on the other side, the pH of the reaction medium can significantly affect the formation and stability of the colored complex (benzoyl hippuric acid) formed during the derivatization of HA with BSC. Changes in pH can impact the ionization state and reactivity of the functional groups in both hippuric acid and BSC. Adding more BSC to the reaction mixture can enhance the derivatization of HA, leading to the formation of more benzoyl hippuric acid. As a result, the concentration of the colored product increases, which generally leads to higher absorbance readings. This is because you have more of the compound responsible for absorbing light in the sample. Lowering the pH of the reaction to a more acidic condition can also enhance the reactivity of BSC and the derivatization reaction. At a lower pH, the functional groups involved in the reaction may be in their more reactive forms, favoring the conversion of HA to benzoyl hippuric acid. This, too, can lead to increased formation of the colored product and higher absorbance readings. So, the combination of increasing the BSC volume and decreasing the pH likely creates a synergistic effect. Both adjustments promote a more efficient reaction, resulting in a higher concentration of the colored compound and, consequently, an increase in absorbance. This suggests that these two factors when optimized together, contribute to the sensitivity and accuracy of the colorimetric detection of hippuric acid.

Figure 4b shows the interaction between sample pH and the volume of methanol. the maximum absorbance occurred by decreasing the pH and the methanol volume. Methanol, as a solvent, can interact with the HA and influence its properties, such as solubility and chemical reactivity. Changes in methanol volume can modify these interactions and impact the overall reaction. In high value of sample pH, increasing the volume of methanol can dilute the reactants, including HA and any other chemicals involved in the colorimetric reaction. This dilution can lower the effective concentration of reactants, making it less likely for them to collide and react to form the colored product.

The interaction between pH and time is shown in Fig. 4c as it is clear, the maximum signal was obtained in 7.0 min of incubation. Chemical reactions have specific rates at which they occur. During the incubation period, the reagents have time to interact and react with HA. The incubation time is optimized to ensure that the reaction proceeds at a rate that allows for the maximum conversion of the analyte into the colored product. Longer incubation times are often needed for reactions with slower kinetics. The incubation time is often optimized to make a balance between the reaction and minimizing the degradation of the colored species. If the incubation time is too long, the colored products could degrade, leading to inaccurate results. The effect of incubation time was evaluated in the ranges of 7–21 min; the findings indicated that high absorbance was recorded within 7 min. The colorimetric reaction may have an optimal pH range at which it proceeds most rapidly and with the highest yield. At this pH, the reaction kinetics are favorable, and the rate of product formation is at its peak. This coincides with the 7-min incubation time, resulting in maximum absorbance.

After evaluating the validity of the model, optimization was performed to obtain the optimum conditions of effective parameters in the determination of HA, including the pH of the sample solution: 3.0, the BSC volume: 0.8 mL, the volume of pyridine: 0.3 ml, methanol volume: 1ml, and incubation time: 7.0 min.

### The analytical performance of the colorimetric method

**Figure 5:**
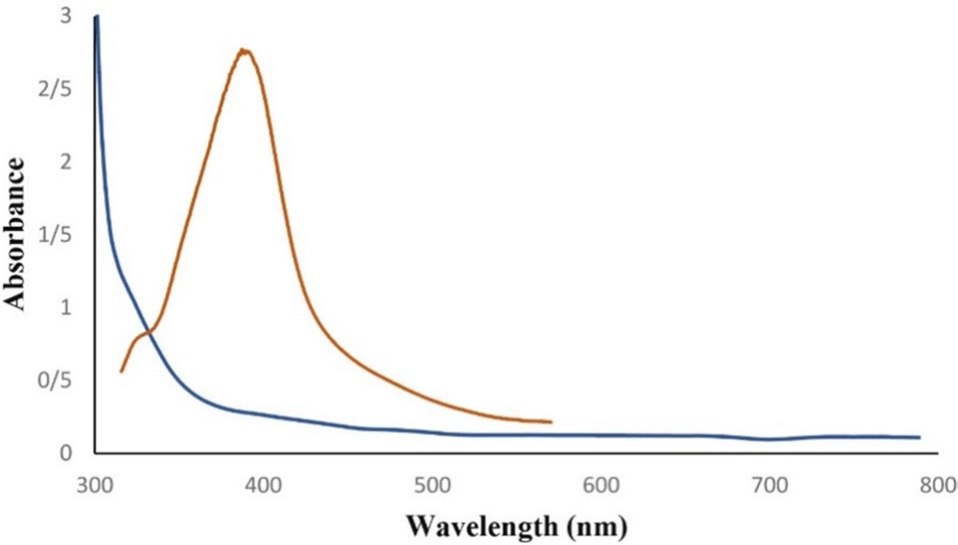
The adsorption spectrum of the urine sample taken from healthy volunteers (**a**), and spiked urine with HA (**b**).

To evaluate the efficiency of the optimized colorimetric method, the analytical performance parameters including the dynamic linear range (DLR), squared correlation coefficients (R^2^), detection limit (LOD), quantification limit (LOQ), precision, and accuracy were evaluated, and are shown in Table 4. The linearity of the colorimetric method was evaluated by spiking five urine samples with increasing concentrations of HA from 6 to 100 mg L^−1^. As presented in Table 4, The absorbance at 410 nm showed excellent linearity with the HA concentration in the range of 6.0–100 mg L^−1^, with R^2^ = 0.97. The LOD was determined using the rule of three times the standard deviation of the blank divided by the slope of the calibration curve. The LOQ is determined by using the rule of ten times the standard deviation of the blank divided by the slope of the calibration curve. The LOD and LOQ were 1.8 mg L^−1^ and 6 mg L^−1^, respectively. The accuracy of the colorimetric method was also investigated in terms of recovery (R%) by spiking the urine samples with HA at five concentration levels (6, 10, 30, 50, and 100 mg L^−1^). The recovery was determined in three replicates based on the optimum conditions and was calculated in the range of 90.5–100.1%. The precision of the method was investigated in terms of the relative standard deviation (RSD) and reported to be less than 5% at n = 3, presenting an acceptable reproducibility. Figure 5 presents the spectra of urine samples with and without spiking HA. As shown in Fig. 5, the resulting spectrum exhibited minimal absorbance at 410 nm. This absence of significant absorbance at 410 nm in the diluted sample indicates that the spectral interference from the inherent yellow color of the urine matrix has been effectively mitigated and controlled.

**Table 4:** Analytical characteristics of the method.

Correlation coefficient (r^2^)	LOD (mg L^−1^)	LOQ (mg L^−1^)	Added (mg L^−1^)	Found (mg L^−1^)	Accuracy	Precision (n = 3)	
					Recovery (%)	Intra-day (RSD %)	Inter-day (RSD %)
0.971	1.8	6	6	5.86	97.6	3.6	4.50
			10	18.76	94.0	2.5	3.45
			30	27.14	90.5	1.7	2.10
			50	50.05	100.1	2.1	2.23
			100	92.80	92.8	4.1	3.5

### Selectivity of the colorimetry assay

**Figure 6:**
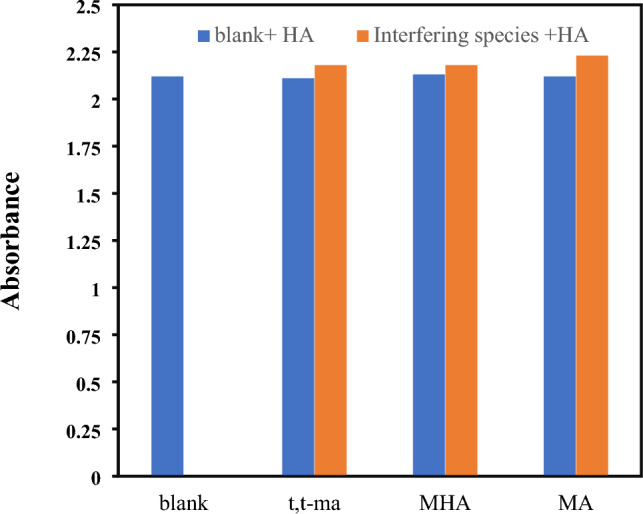
Selectivity of the proposed colorimetric method. The UV–Vis absorbance at 410 nm in the presence of HA (10 ppm) and other interfering substances (50 ppm).

There are several potential interfering substances, such as trans, trans muconic acid (t,t-MA) as an exposure biomarker of benzene, methyl hippuric acid (MHA) as a biomarker of xylene, and mandelic acid as a biomarker of ethyl benzene in urine samples of workers who exposure to HA. To evaluate the selectivity of the proposed method, in the presence of HA, the above interferers were added separately to the system and the resulting absorbance at 410 nm was recorded. The blank sample + HÀ was considered as a control sample. In these experimental tests, the concentrations of these interfering substances were fivefold higher than that of HA (10 µg mL^−1^). As shown in Fig. 6, the interfering spices did not significantly affect the experimental results for the detection of HA. It indicates that the selective colorimetric response was induced by only the addition of HA.

### Correlation of HPLC and colorimetric methods in the real urine sample

**Figure 7:**
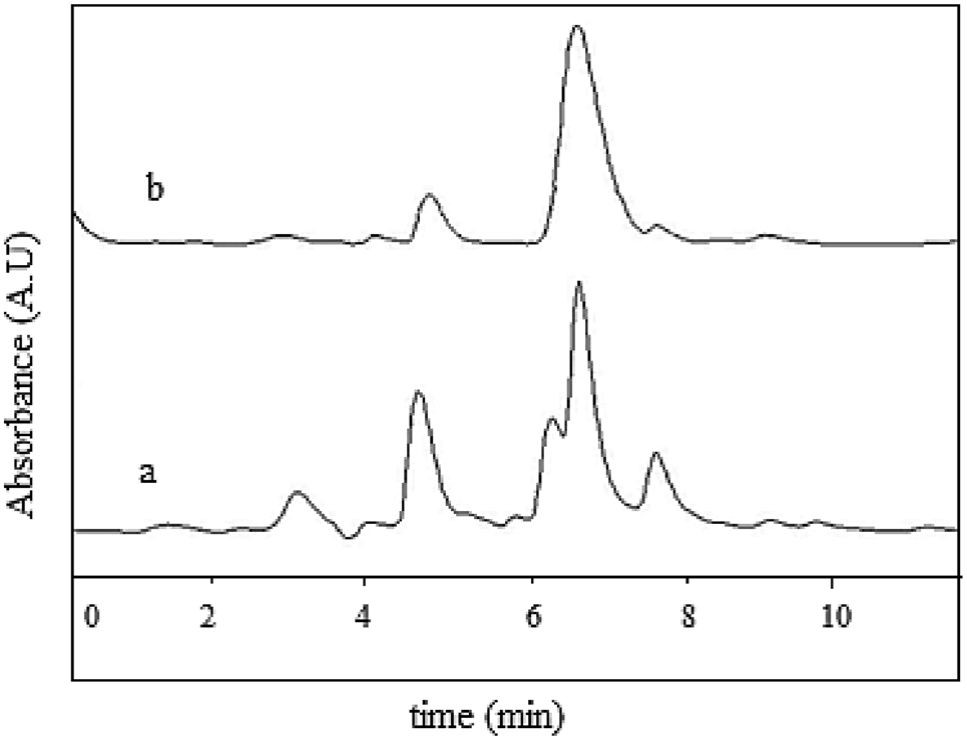
Chromatograms of a real urine sample taken from a worker before (**a**) and after (**b**) applying sample preparation.

The optimized procedure was used for the determination of HA in urine samples by colorimetric method, and compared with the usual HPLC methods. We had two groups of urine samples including 10 samples taken from unexposed healthy volunteers spiked with a specific amount of HA (6.6 to 100 mg L^−1^), and 10 urine samples taken from workers who were exposed to toluene in a petrochemical industry. A chromatogram of a real urine sample taken from a worker before and after applying sample preparation is shown in Fig. 7. As shown various interreferences were presented before sample pretreatment.

Creatinine correction was performed because differences in urine dilution can impact the accuracy of HA determination. The levels of creatinine in urine were determined by using Jaffe's method based on spectrophotometry^33^.

The average level of HA was 827.7 mg/g creatinine in HPLC analysis, and 819.5 mg/g creatinine for the colorimetric method. The average concentration of HA in toluene of exposed workers was 362.9 mg/g creatinine by HPLC analysis, and 362.2 mg/g creatinine by colorimetry method, respectively. The overall results showed a good agreement between our method in the real sample and the HPLC standard method for the determination of HA. As seen in the tables, for higher concentrations of HA, the level of agreement between the results obtained from the two methods is higher (Tables 5, 6). By conducting linear regression analysis on the results, it was found that for concentrations higher than 500 mg/g creatinine, there is a significant linear relationship between the results.

**Table 5:** The concentration of HA in urine taken from unexposed healthy volunteers spiked with a specific amount of HA (6.0 to 100 mg L^−1^).

Sample no	Creatinine (mg/dl)	HA concentration		
		HPLC method (mg/g creatinine)	Colorimetric method (mg/g creatinine)	Residual^a^
#1	150	923	879	− 44
#2	134	650	666	16
#3	102	1010	1122	112
#4	213	575	498	− 77
#5	98	308	315	7
#6	87	1420	1485	65
#7	83	409	412	3
#8	101	803	821	18
#9	74	1109	978	− 131
#10	123	1070	1024	− 46

^a^Difference between colorimetric and HPLC methods.

**Table 6:** The concentration of HA in urine samples taken from exposed workers.

Sample no.	Creatinine (mg/dl)	HA concentration		
		HPLC method (mg/g creatinine)	Colorimetric method (mg/g creatinine)	Residual^a^
#1	102	367	323	− 44
#2	100	115	120	5
#3	99	126	130	4
#4	83	543	521	− 22
#5	87	210	199	− 11
#6	93	412	419	7
#7	97	619	621	− 2
#8	81	308	298	− 10
#9	93	313	377	64
#10	103	626	614	− 12

^a^Difference between colorimetric and HPLC methods.

### Comparison of the proposed technique with previously published techniques

A comparison between LOD, DLR, and RSD, as well as the extraction solvents and analysis time of the proposed techniques is presented in Table 7. Most of the previous methods were developed based on LPME or SPME, and chromatographic analysis. The number of three colorimetric assays have been previously proposed for detecting HA in urine samples. As shown in Table 7, all of the HPLC methods need an additional step of sample preparation, involving extraction and sometimes derivatization. These steps require higher consumption of solvents, especially in liquid–liquid extraction techniques. In contrast, colorimetric methods often involve simpler sample preparation and reactions, resulting in relatively lower solvent consumption. In addition, the colorimetric methods are more cost-effective due to the availability of simple and inexpensive reagents. Therefore, colorimetric methods are typically much faster than HPLC techniques because chromatography often requires more extensive sample preparation, including extraction, and derivatization, and this can be time-consuming.

**Table 7:** Comparison between the proposed method for the determination of HA with other previous studies.

Method	Sample preparation	Extraction solvents/sorbent	Additional solvent	LOD (mg L^−1^)	DLR (mg L^−1^)	RSD	Time (min)	Ref.
HPLC–UV	LLE	Ethyl acetate	–	4	10–1000	5.3–6.1	20	^30^
HPLC–UV	DLLME	4-Chlorophenol	Acetonitrile	0.008	0.024–20	< 4.5	–	^21^
HPLC–UV	D-μ SPE	mesoporous silica (KIT-6) sorbent	NH_4_OH- methanol	0.0005	0.001–1	< 6.0	–	^1^
HPLC–UV	MEPS	2,4-Dihydroxybenzoic acid, formaldehyde	Acetic acid, ammonia	0.01	5–1000	2.09–3.41	–	^23^
HPLC–UV	DES-DLLME	ChCl:4-Chlorophenol	Acetonitrile	0.008	0.024–20/0	< 4.5	–	^21^
Spectrophotometry	–	–	p-dimethyl-amino benzaldehyde, pyridine	2000	500–2000	1.3–2.7	90–120	^35^
Spectrophotometry	–	–	Benzene sulfonyl chloride (BSC), acetic acid	125	125–2000	–	30	^36^
Spectrophotometry	–	–	*p*-toluenesulfonyl chloride,	10	10–180	0.82–8.7	2	^29^
Spectrophotometry	–	–	benzenesulfonyl chloride (BSC), pyidinene, methanol	1.8	6–100	< 5	10	Present study

LLE: liquid–liquid extraction, DLLME: dispersive liquid–liquid microextraction, D-μ SPE: dispersive micro solid phase extraction, LOD: limit of detection, DLR: dynamic linear range.

A comparison between the analytical performance of the proposed methods revealed that the HPLC-based methods exhibited a lower LOD for HA determination compared to colorimetric methods. The lower LOD indicated that the HPLC methods are more sensitive in detecting lower concentrations of HA in urine samples. However, the LOD achieved through the colorimetric method in our study has proven to be well-suited and reliable for occupational exposure levels. The sensitivity of this method is sufficient to detect HA in the range of occupational exposure limits. Other analytical performance parameters such as DLR and RSD are in an acceptable range in both colorimetric and HPLC methods.

A comparison between the existing colorimetric methods showed that the overall time of the analysis, the LOD and DLR values, and the type of the solvents are different between methods. In the older study, the analysis time was considerably high^24^. However, in two subsequent studies, there has been a notable reduction in the analysis time from 120 min to 30 and 2 min^29,34^. The overall analysis time in our study was 10 min, which is an acceptable time compared with HPLC techniques and older colorimetric methods. Compared with other existing colorimetric methods, our study exhibited a lower LOD, indicating a more applicable method for the determination of a lower concentration of HA in occupational exposure. In general, our results demonstrated that the proposed colorimetric method will be capable of measuring HA levels in urine samples from workers exposed to toluene within a short time, with an acceptable limit of detection.

## Remarkable conclusion

In the assessment of HA levels in occupational settings, the choice of analytical method plays an important role in ensuring the health and safety of exposed workers. Our study aimed to evaluate the correlation between two analytical techniques, colorimetric methods, and HPLC, in determining HA concentrations. By improving the efficiency of toluene exposure assessment, our study contributes to overall public health. Minimizing toluene exposure risks can prevent various health issues, such as respiratory problems, neurological effects, and potential long-term outcomes. By applying a simple, fast, and inexpensive method, we could conduct the regular screening program several times in a year. This could be particularly beneficial in regions or industries with limited resources, extending the reach of exposure monitoring to a broader population.

### Correlation in low concentrations

In the initial phases of our study, where HA concentrations were low, the correlation between the colorimetric methods and HPLC was less than satisfactory. This observation is consistent with the inherent limitations of colorimetric techniques, which may struggle to provide accurate results when dealing with trace amounts of a target compound. The sensitivity and specificity of colorimetric assays can vary depending on factors such as reagent quality and the presence of interfering substances, and this can lead to discrepancies in results.

### Improved correlation at higher concentrations

In higher concentrations of HA, we observed a substantial improvement in the correlation between the colorimetric methods and HPLC. The reasons behind this shift are multifaceted. Colorimetric methods, while less sensitive than HPLC, tend to perform more reliably in the quantification of compounds when they are present in higher concentrations. The reactions upon which colorimetry relies become more pronounced, resulting in more accurate and consistent results.

### Implications for occupational exposure assessment

This study holds significant implications for occupational exposure assessment. In many workplace environments, the levels of HA exposure can vary widely, often dependent on the nature of the tasks performed and the extent of protective measures. In situations where occupational exposure levels are consistently high, such as in certain industrial processes or specific job roles, our findings suggest that colorimetric methods may offer a pragmatic alternative to HPLC for routine monitoring.

### Balancing precision with practicality

While HPLC remains the gold standard for precision and accuracy, it is essential to strike a balance between precision and practicality in the context of occupational health and safety. Colorimetric methods are cost-effective, rapid, and well-suited for high-throughput screening, making them a valuable tool for monitoring workers' exposure when higher concentrations of HA are expected. By utilizing colorimetry in these scenarios, organizations can streamline their monitoring processes and allocate resources more efficiently. The innovation of the present study was optimizing a colorimetric method to obtain a simple and fast analysis for HA. In a specific analytical condition, the speed of the reaction between reagents and analytes may be too fast. We demonstrated that the rate of product formation in the colorimetric reaction of HA by benzene sulfonyl chloride is maximum in the acidic condition. This coincides with the 7-min incubation time, resulting in maximum absorbance. Therefore, this method seems to be an alternative to the HPLC method for HA determination. Our study evaluates the dynamic relationship between HA concentration levels and the correlation between colorimetric methods and HPLC. While the correlation may be less robust at lower concentrations, it notably improves at higher concentrations. This finding suggests that, in occupations where exposure levels are consistently high, colorimetric methods can be a practical and cost-effective choice for routine monitoring.

### The limitations and strengths of the present study

The primary strength of the present study is associated with the speed of the colorimetric assay, providing results in a shorter time compared to traditional methods like HPLC. The affordability of the colorimetric assay enhances its practicality for routine monitoring. the study’s comparison with HPLC demonstrates the feasibility and reliability of the method, offering a practical alternative without compromising accuracy. The overall limitations of the colorimetric methods may relate to the lack of sensitivity for measuring low concentrations of analyte.

## Data Availability

Most of the required data is presented in the manuscript. Other datasets generated during and/or analyzed during the current study are available from the corresponding author upon reasonable request.
